# Artificial Intelligence-Based Hole Quality Prediction in Micro-Drilling Using Multiple Sensors

**DOI:** 10.3390/s20030885

**Published:** 2020-02-07

**Authors:** Jitesh Ranjan, Karali Patra, Tibor Szalay, Mozammel Mia, Munish Kumar Gupta, Qinghua Song, Grzegorz Krolczyk, Roman Chudy, Vladislav Alievich Pashnyov, Danil Yurievich Pimenov

**Affiliations:** 1 Department of Mechanical Engineering, Indian Institute of Technology Patna, Patna-801103, India; jitesh4u89@gmail.com; 2 Department of Manufacturing Science and Engineering, Budapest University of Technology and Economics, H-1111 Budapest, Hungary; szalay@manuf.bme.hu; 3 Department of Mechanical Engineering, Imperial College London, Exhibition Rd., London SW7 2AZ, UK; m.mia19@imperial.ac.uk; 4 Key Laboratory of High Efficiency and Clean Mechanical Manufacture, Ministry of Education, School of Mechanical Engineering, Shandong University, Jinan 250100, Chinassinghua@sdu.edu.cn (Q.S.); 5 Faculty of Mechanical Engineering, Opole University of Technology, 76 Proszkowska St., 45-758 Opole, Poland; g.krolczyk@po.opole.pl (G.K.); r.chudy@po.edu.pl (R.C.); 6 Department of Automated Mechanical Engineering, South Ural State University, Lenin Prosp. 76, Chelyabinsk 454080, Russia; alich74@rambler.ru (V.A.P.); danil_u@rambler.ru (D.Y.P.)

**Keywords:** micro drilling, vibration, cutting force, wavelet packet, adaptive neuro fuzzy inference system

## Abstract

The prevalence of micro-holes is widespread in mechanical, electronic, optical, ornaments, micro-fluidic devices, etc. However, monitoring and detection tool wear and tool breakage are imperative to achieve improved hole quality and high productivity in micro-drilling. The various multi-sensor signals are used to monitor the condition of the tool. In this work, the vibration signals and cutting force signals have been applied individually as well as in combination to determine their effectiveness for tool-condition monitoring applications. Moreover, they have been used to determine the best strategies for tool-condition monitoring by prediction of hole quality during micro-drilling operations with 0.4 mm micro-drills. Furthermore, this work also developed an adaptive neuro fuzzy inference system (ANFIS) model using different time domains and wavelet packet features of these sensor signals for the prediction of the hole quality. The best prediction of hole quality was obtained by a combination of different sensor features in wavelet domain of vibration signal. The model’s predicted results were found to exert a good agreement with the experimental results.

## 1. Introduction

Due to the emergence of the miniaturization of products in aerospace, biomedical, communication, electronics and automotive industries, the low cost of such miniaturized products with their best-of-class quality makes them suitable for extensive use of micro-machining processes. The adopted micro-machining processes include microturning [1,2], micromilling [3,4,5,6], microdrilling [7,8]. Micromachining is generally defined as the machining process that produces miniature component or feature in of the range of 1µm to 999 µm [9]. Several non-conventional methods such as micro electric discharge machining (μEDM) [10,11,12], laser micromachining [13,14], ultrasonic [15,16,17], electron beam machining (EBM) [18,19], etc. are mostly used to produce miniaturized product/feature this range. However, these methods are inferior to conventional machining due to some constraints like relevance only to certain shapes and non-suitability of a wide range of material types. On the other hand, mechanical micromachining methods which can produce any arbitrary shapes and are applicable to almost each type of material have wide acceptance to fabricate miniaturized products or micro-features [20]. The commercial micro tools are now widely manufactured, nevertheless it is difficult to measure wear and determine the fractures of such small size tools. Therefore, tool-condition monitoring of micromachining is crucial to improve the productivity and reliability of the process [21,22]. Effective monitoring system can provide precise estimation of tool wear with diverse cutting conditions and also provides operational safely for the operator [23,24,25].

Indirect monitoring is based on different cutting parameters by the different sensor signals such as thrust force [26,27,28], torque [29], current [30,31], spindle power [32], computer vision [33,34], vibration [35,36], acoustic emission [37], etc. These indirect parameter measurements are related to the tool condition. Through these signals, we analyze the cutting condition as well as tool condition by various signal processing techniques namely time domain signal, fast Fourier transform, wavelet packets transform and other higher-order transformations [38,39]. Kim et al. [26] used a force signal to monitor tool condition during micro hole drilling. Torque signal has also been extensively used for monitoring tool condition. In a drilling operation, torque increases as the tool wear increases. Oh et al. [30] investigated drill wear reduction in macro-drilling using a torque control method though measurement of a spindle motor current. Vibration signals are widely used for condition monitoring. The main advantages of the study of vibration signal are its ease of implementation as compared to other signals and no modification is required to the workpiece and fixture. Huang et al. [35] used vibration signals to monitor tool condition. Input impedance of driving motor measure features the same principle as torque, because they both focus on the amount of power used in the machining process. Fu et al. [40] used input impedance of driving motor to monitor tool condition during micro drilling. Kim et al. [26] investigated drill wear on the basis of spindle motor power. However, for effective monitoring of tool conditions in the complex cutting mechanism, a sensor fusion is needed that can eliminate the problems of the limited bandwidth of individual sensor [25,41,42]. Segreto et al. [41] analyzed machined surface integrity through multiple sensor monitoring based on cutting force, acoustic emission and vibration signal analysis. Plaza et al. [42] also proposed multi-sensor data fusion for in-process quality control of machining based on cutting force, vibration and acoustic emission signals. Similarly, Malekian et al. [25] showed improvement of tool-wear monitoring in mechanical micro-milling using multiple sensor data fusion based on all the aforementioned three sensor signals.

Machining operations, specifically, the micro drilling process, involve models which are highly non-linear. Analytical models divulge better results of wear progression mechanism, but sometimes they are less accurate because of simplifications due to several assumptions. Artificial intelligence-based systems such as the artificial neural network (ANN) [43,44,45], the convolutional neural network (CNN) [46,47] and the adaptive neural fuzzy inference system (ANFIS) [48] are considered feasible, consistent and smart approaches for predicting the condition of cutting tools. These techniques can realize non-linear relations among machining parameters, process signals and tool states for their high fault tolerance and adaptability features. Rahman et al. [43] applied ANN in turning operations for monitoring wear, chip breaking and tool chatter. Sharma et al. [44] applied ANN for predicting tool wear from cutting forces, vibrations and acoustic emission signals. Patra et al. [45] also used an ANN-based model for micro-drill condition monitoring by using vibration signals. In recent works, Wang et al. [46] and Cao et al. [47] showed that convolutional neural network can be more effective and faster than the conventional networks like ANN, support vector machine (SVM), etc. for prediction/identification of system performances through multi-sensor data.

The application of ANFIS is also widespread; it is a hybrid modelling technique that comprises fuzzy logic and ANN technique. In this hybrid technique, a fuzzy model is first developed using the rules extracted from the input output data of the system in hand. Then, the ANN is used to fine tune the rules of the fuzzy model and the final ANFIS model of the system is obtained. Malekian et al. [25] used features of acoustics emission, force and acceleration sensors, cutting parameters and the tool edge radius value as input to the neuro-fuzzy algorithm to estimate tool conditions (good, average, bad). Beruvides et al. [48] used ANFIS for predicting tool wear in micro-drilling.

For an effective ANFIS-based tool condition monitoring system, it is necessary to use data of process signals for a number of drilled holes up to the drill breakage for a variety of cutting conditions. In this work, the vibration signals and cutting-force signals have been applied individually and in combination to determine their effectiveness for tool condition monitoring applications in terms of hole quality and also to determine best strategies for tool condition monitoring. The time domain and wavelet features of these signals have been used in ANFIS models to predict the hole quality. Furthermore, the hole quality prediction by wavelet features of vibration signal was compared with the same by the time domain features.

The purpose of this article is to predict the quality of the holes obtained in micro-drilling through monitoring the condition of the tool and detecting the tool breakage by thrust force, torque and vibration signals using artificial intelligence.

## 2. Materials and Method

**Figure 1:**
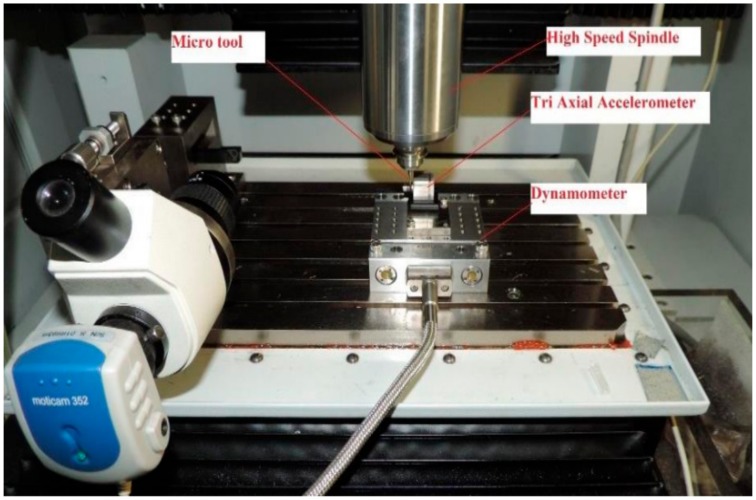
Experimental setup for micro-drilling.

Micro-drilling experiments were conducted on CNC micro machining centre (Model DT110, Mikrotools Pte Ltd, Singapore) as shown in Figure 1. A triaxial accelerometer and a Kistler Minidyn 9256C2 dynamometer (Aachen, Germany) were used to record the vibration signal, force signal and torque signal. The sampling frequency of 10,000 Hz was set in NIPXI 1052 data acquisition system (National Instruments, Austin, TX, USA) for obtaining vibration signals. The force and torque were acquired by charge amplifier 5070A (Kistler, Aachen, Germany) and dynoware software with a sampling rate of 1000 samples/s. Solid carbide drills (manufactured by Seco Tools GmBH, Fagersta, Sweden) with diameter of 0.4 mm were applied on austenitic stainless steel workpiece to make blind hole of 1.0 mm depth.

The experimental conditions are shown in Table 1. As the feed value is very small (in micrometer range) in microdrilling, high spindle speed generally is used to reduce the machining time for hole making. Anand et al. [49] used very high cutting speed (15-40 m/min corresponding to 10,000–25,000 rpm) and showed that the specific thrust force value does not vary much by increasing the cutting speed from the recommended cutting speed range of the manufacturer. Therefore, this work used cutting speed range of 12.8–24 m/min (10,186 rpm to 19,099 rpm).

There is also another reason for using higher cutting speed. The higher cutting speed (spindle rpm) was shown to accelerate tool wear [50]. Therefore, by using higher cutting speed, we could get increased cutting force and vibration with less number of drilled hole. This helps us to develop the model will less number of experiments.

## 3. Results and Discussion

### 3.1. Signal Analysis

#### 3.1.1. Time Domain Features of Process Signals

**Figure 2:**
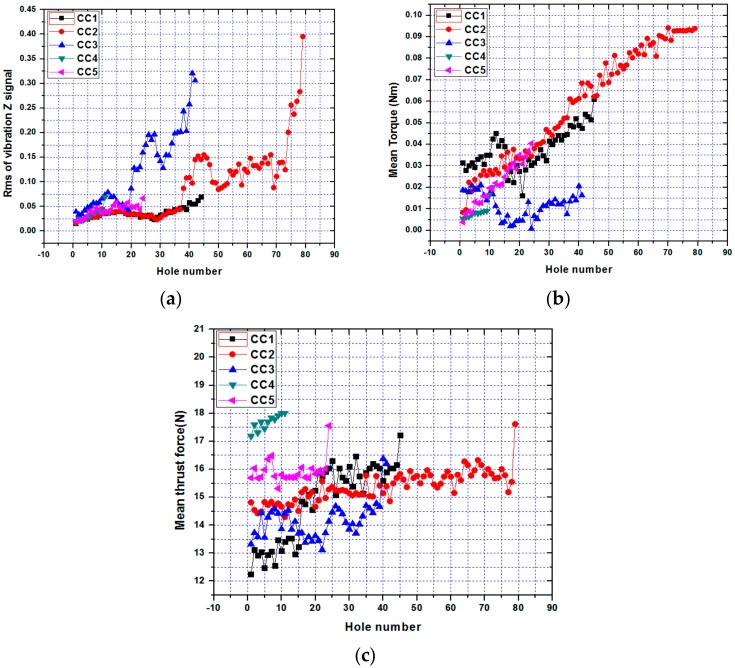
The variations of (**a**) vibration, (**b**) thrust force and (**c**) torque features with respect to the hole numbers.

The time domain features extracted from different process signals are mainly arithmetic mean, root mean square (RMS) value, kurtosis, and standard deviation [51]. Among these features, RMS value is the most common one that represents most of the signal energy. The time domain features extracted from vibration signal is root mean square (RMS), whereas, mean value of thrust force and mean value of torque are the two other time domain features. The variations of vibration, thrust force and torque features with respect to the hole numbers before the tool breakage of different cutting condition are shown in Figure 2a–c. From Figure 2a. it is clearly seen that at constant feed rate *f_r_* = 0.003 mm/rev and different spindle speeds *n* = 14,186 rpm (CC5) and *n* = 19,099 rpm (CC4), an increase in rms of vibration Z signals is observed with respect to lower spindle speed *n* = 10,186 rpm (CC1). A similar trend with constant spindle speed *n* = 14,186 rpm and different feed rates *f_r_* = 0.002 mm/rev (CC2) and *f_r_* = 0.003 mm/rev (CC5) is also observed. In addition, for feed rate *f_r_* = 0.001 mm/rev, spindle speed *n* = 19,099 rpm (CC3), the rms of vibration Z signals increase after 20 number holes and increase sharply after 30 number holes. For feed rate *f_r_* = 0.002 mm/rev, spindle speed *n* = 14.186 rpm (CC3), rms of vibration Z signals increase after 37 number hole and increase sharply after 73 number hole. These phenomena can be explained by the growth of flank wear micro drill. Figure 2b shows that the mean torque increases with increasing hole number. However, with increasing spindle speed from *n* = 10,186 rpm (CC1), *n* = 14,186 rpm (CC5) to *n* = 19,099 rpm (CC4) and constant feed feed rate *f_r_* = 0.003 mm/rev, we observe a decrease in mean torque. Moreover, an increase in feed rate from *f_r_* = 0.002 mm/rev (CC2) to *f_r_* = 0.003 mm/rev (CC5) and a constant spindle speed *n* = 14,186 rpm on the contrary leads to an increase in mean torque. Figure 2c shows that mean thrust force tends to increase with increasing hole, but with less intensity compared to mean torque. However, with increasing spindle speed from *n* = 10,186 rpm (CC1), *n* = 14,186 rpm (CC5) to *n* = 19,099 rpm (CC4) and constant feed feed rate *f_r_* = 0.003 mm/rev, we observe increase in mean thrust force. A similar trend at feed rate from *f_r_* = 0.002 mm / rev (CC2) to *f_r_* = 0.003 mm / rev (CC5) and a constant spindle speed *n* = 14,186 rpm leads to an increase in mean thrust force. With increasing feed rate from *f_r_* = 0.001 mm/rev (CC3) to *f_r_* = 0.003 mm/rev (CC4) and a constant spindle speed *n* = 19,099 rpm, the trend also increases in mean thrust force.

It can be seen that amplitudes of these signals increase non-linearly with the number of holes drilled. With increase in hole number, tool wear increases that contributes to increase RMS and mean values of these signals [23,45] shown in the figures. It can also be seen that RMS values of vibration signals increase suddenly just before the breakage of the tool [45]. On the other hand, the magnitudes of torque and thrust signals do not show any sudden change prior to tool breakage [23].

#### 3.1.2. Wavelet Packet Features of Vibration Signals

As mean and RMS values of time domain features are determined by considering the whole signals, these are likely to be affected by the noise and disturbance present in the drilling process. To eliminate the noise and disturbances, many attempts have been made earlier to extract features in the frequency domain and the time-frequency domain [48,52]. Among different time-frequency domain signal processing methods, the most common is the wavelet packet transform which can overcome the poor time localization of a fast Fourier transform (FFT) and loss of important information due to not consideration of the detail part in case of a discrete wavelet transform (DWT). In this work, the wavelet packet transform is performed to decompose the vibration signal (up to 5 kHz) into 8 frequency bands (8 wavelet packets) up to level 3 [53]. These packets are denoted as packet (3,0), packet (3,1), packet (3,2), packet (3,3), packet (3,4), packet (3,5), packet (3,6) and packet (3,7) corresponding to frequency range of (0–0.625 kHz), (0.625–1.25 kHz), (1.25–1.875 kHz), (1.875–2.5 kHz), (2.5–3.125 kHz), (3.125–3.75 kHz), (3.75–4.375 kHz) and (4.375–5 kHz), respectively.

Wavelet packet coefficients are estimated using Daubechies 8 wavelet function. Wavelet packet feature ($C_{\mathrm{ij}})$ which is the RMS value of the wavelet coefficients for a packet is determined to relate the condition of the tool at different cutting conditions. Here, i is the decomposition level and j stands for the packet number at the ith level.

**Figure 3:**
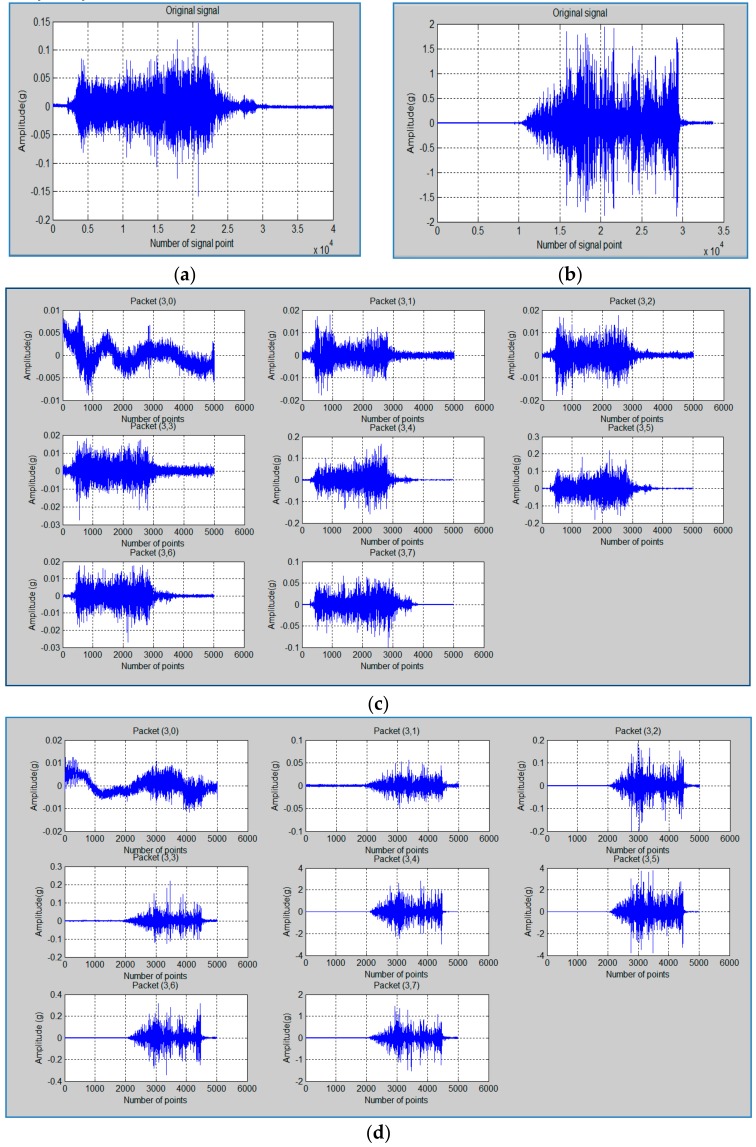
Time signal for (**a**) 1st hole and (**b**) 79th hole of vibration. The wavelet packet signal for (**c**) 1st hole and (**d**) 79th hole of vibration. Data taken in Z direction, and the signal is of cutting condition CC2.

Figure 3a,b shows the time signal comparison of the first hole and 79th hole of the cutting condition (CC2). It can be observed that the amplitude of vibration for the 79th hole is very high in comparison with the same for the 1st hole as tool wear becomes severe for later case. This leads to tool failure after the 79th hole. The observation is in the similar line to that of trends reported earlier [45]. Figure 3c,d present all the 8 wavelet packets of 1st hole and the 79th hole (before the tool breakage), respectively. It can be observed that in packet (3,4), (3,5) and (3,7) the amplitude variation was very high compared with the 1st hole packet; however, the other packet’s amplitude variation is comparably very low.

**Figure 4:**
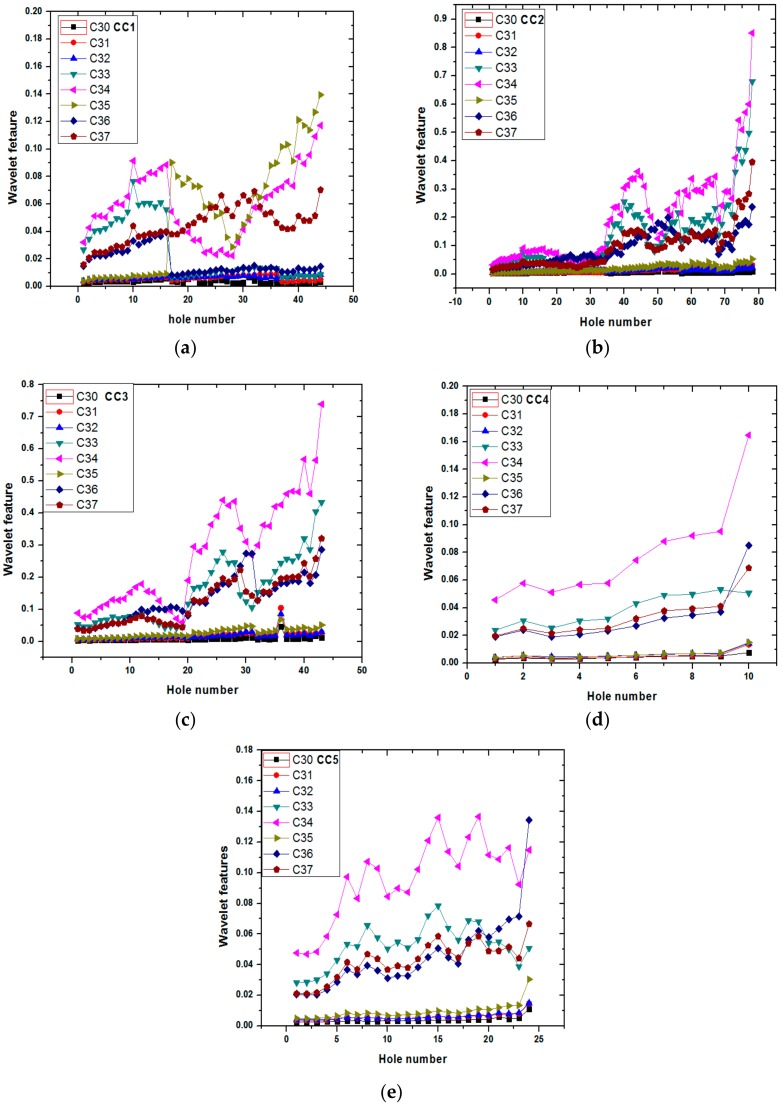
Wavelet packet features for different cutting conditions (**a**) CC1; (**b**) CC2; (**c**) CC3; (**d**) CC4; (**e**) CC5 from 1^st^ hole to last hole before drill breakage.

Each wavelet packet carries information on the vibration signal in terms of wavelet coefficients. But some of the packets also contain noise or less important information depending on the process dynamics [50]. Figure 4a–e shows variations of wavelet coefficients with hole number for all 5 cutting conditions. In all 5 plots, 3 or 4 frequency bands such as C34, C35, C37 have non-linearly increasing trends as compared to other wavelet packets of the first hole to till brakage hole. The less amplitude packets contain noise and are not affected by the tool condition. Features that show increasing trends have been taken as input of soft computing techniques (i.e., ANFIS).

### 3.2. Hole Quality

**Figure 5:**
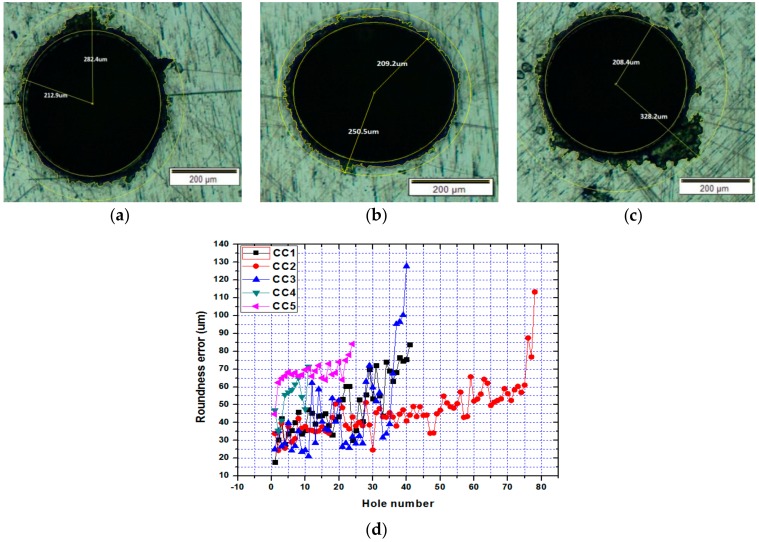
Images of drilled holes (**a**) no.1, (**b**) no. 40, and (**c**) no. 79 for the cutting condition 2 (CC2); (**d**) variations of roundness error with respect to the hole numbers with different cutting conditions.

The predictability and effectiveness of the two models are based on the hole quality. Typically, the hole roundness error is used as a measure of hole quality in drilling [54]. To determine the roundness error, maximum circles are inscribed inside and outside the hole profile and their radii are defined as outer radius and inner radius of the hole. The difference of outer radius and inner radius of the hole is called roundness error [55]. For each cutting condition, the images of all drilled holes before the drill breakage were captured by a high-resolution microscope, and its outer and inner radius of each hole are determined by digimizer software tool as shown in Figure 5a–c for the 1st, 40th and 79th holes of cutting condition CC2. The hole roundness error is calculated for individual hole and plotted against hole number for different cutting conditions as shown in Figure 5d. Comparing the influence of various values of cutting speeds and feeds from Figure 5 the following relationships are visible. With a constant feed rate *f_r_* = 0.003 mm/rev, with increase of cutting speed *n* = 14,186 rpm (CC5) and *n* = 19,099 rpm (CC4) we have an increase in the average roundness error compared to that of cutting speed of 10,186 rpm (CC1).Similarly, with a constant spindle speed of n = 14,186 rpm and different feed rate *f_r_* = 0.002 mm/rev (CC2) and *f_r_* = 0.003 mm/rev (CC5), we get the increase of average roundness errors with increase in feed. A similar situation is observed with a constant spindle speed of *n* = 19,099 rpm and different feed speed *f_r_* = 0.001 mm / rev (CC3) and *f_r_* = 0.003 mm / rev (CC4). In addition, for CC2 and CC5 it is clearly seen that with a hole number of 75 and 36, respectively, the average roundness error increases sharply. This can be explained by the development of catastrophic wear of the micro drill.

The roundness error value increases non-linearly with the drilled hole numbers. The nonlinear increase of roundness error with increasing hole number is related to the increase of cutting forces and vibration magnitudes. Increase in tool deflection due to change in cutting forces and vibration amplitude increases the roundness error [40]. Furthermore, it can be observed that the trend of roundness error is similar to that of the vibration with respect to the drilled hole number.

### 3.3. Tool Condition-Monitoring Based on Hole Quality Prediction Using Multiple Sensors

#### 3.3.1. Adaptive Neuro Fuzzy Inference System (ANFIS) Model for Time Domain Signals

**Figure 6:**
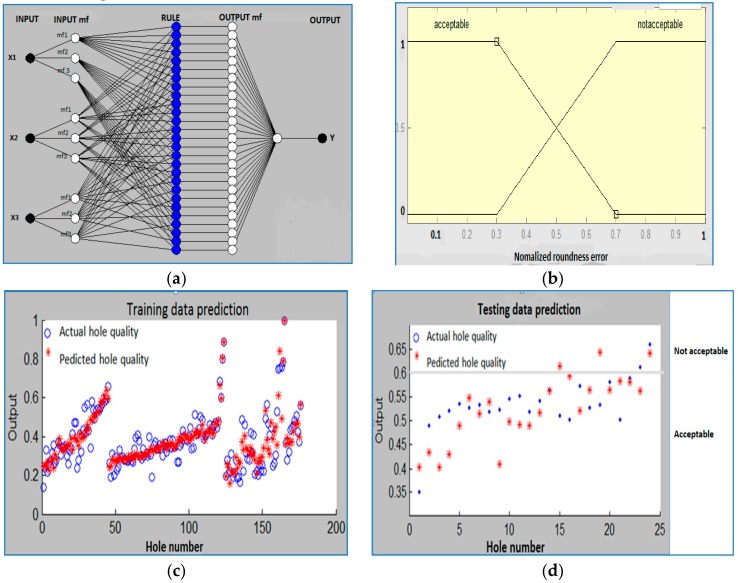
(**a**) Different input, different membership value, rules and combined output architecture; (**b**) Trapezoidal function of normalized hole quality with two membership function; (**c**) Prediction accuracy for training data; (**d**) Prediction accuracy for testing data.

As the hole roundness error depends on the tool condition and cutting conditions, prediction of hole quality through input process signals (vibration, thrust and torque) at any cutting condition can tell the cutting tool condition. The decision can be taken whether the tool is still acceptable or not based on the hole quality. An adaptive neuro-fuzzy inference system (ANFIS) has been introduced to relate input process signals with hole quality parameter. The Matlab ANFIS tool has been used to develop the neuro-fuzzy network. In ANFIS model, fuzzy inference system is superimposed to the neural network. The neural network layer has input, output and a hidden layer. The hidden layer is imposed by fuzzy inference system (i.e., membership function layer and rule-based layer) and an activation function. Features extracted from different sensors and the cutting conditions were used as the inputs to this ANFIS to determine the output parameter, i.e., hole quality. Figure 6a shows the inputs, membership values, rules and combined output architecture of ANFIS model. Here, input parameters X1, X2 and X3 are the mean thrust (Fz), mean torque (Mz) and RMS vibration (Az) from time domain signals.

For training, data from sensor features, i.e., RMS value of vibration signal, mean value of thrust force and torque at 4 cutting conditions (CC1 to CC4 of 175 holes) have been employed. For testing, data with same sensor features from another cutting condition data (CC5 of 24 holes) have been used. Hole quality value could be assigned as an output. Based on hole-quality prediction, we identified the tool condition as ‘acceptable’ or ‘not acceptable’. From Figure 5, it can be observed that roundness errors of the initial drilled holes at different cutting conditions remain in the range of 30–40 μm, similar to the errors reported by Anand and Patra [54] for microdrilling with fresh cutting tools. Though with increase in hole number the roundness error increases, the value remains within 70 μm for most of cutting conditions for most of the time. Thereafter, we observe a sudden increase of roundness error till the breakage of the microdrill. Therefore, hole roundness error has been considered ‘acceptable’ and ‘not acceptable’, corresponding to hole roundness less than 70μm, and greater than 70 μm, respectively. Normalization of both input and output parameters have been conducted and these parameters lie between 0 and 1. A trapezoidal membership function has been chosen for both input and output. Figure 6b define the acceptable and not acceptable hole quality with intersection regions. The presented results can help the operator or the control system to take proper tool changing decisions.

Table 2 lists the combinations of the sensor features, taken for training and testing. The minimum mean training error was 0.0639 for the data set with combination of all force, torque and vibration signal. The corresponding testing error was found to be 0.07037. Figure 6c,d compares the normalized predicted hole quality with the actual hole quality (roundness error) using training and testing data, respectively, obtained by combining mean thrust (Fz), mean torque (Mz) and RMS vibration (Az). Figure 6d also shows the hole quality of acceptable and not acceptable values for the testing data against the hole number. The deviation of some of these data comes due to the non-linear feature of data. Nonlinear features of the data arise due to the uncertainty and complexity of the micromachining process. So we developed a model based on acceptable hole quality. Even though we found deviations of some of the data points, the mean testing error of the selected multi-sensor data fusion approach is low and acceptable. Among the employed strategies of hole-quality prediction, the best performance was given by fusion of different sensor signal; this is due to fact that it takes a wide range of signal bandwidth. At a wide range of bandwidth, the features extracted are more accurate and noise free.

#### 3.3.2. ANFIS Model Using Wavelet Packet-Based Vibration Features

An ANFIS model has also been developed where the inputs were high sensitive wavelet features of Z direction vibration signal at different cutting conditions and the output was the hole quality. The structure of the wavelet packet feature based ANFIS is similar to that shown in Figure 6a. For training, data of 3 highly sensitive wavelet features (C34, C35, C37) of vibration signals for 4 cutting conditions of 176 holes have been considered. For testing, data with same wavelet features of 24 holes of another cutting condition’s data (CC5) have been employed. In this approach also the normalized values between 0 and 1 are used for all input and output parameters. After training, the ANFIS was ready to estimate tool condition based on hole-quality prediction with the testing data. By ANFIS model, the mean training error and the testing error of vibration signal was found as 0.054371 and 0.080652. Table 3 presents the performance comparison of ANFIS models using RMS vibration features and wavelet packet features of vibration signal in Z direction. The errors of training and testing steps are less in a wavelet packet approach as compared to a time domain approach.

## 4. Conclusions

This work develops the indirect tool-monitoring techniques for monitoring tool condition and detecting tool breakage based on hole quality. For this purpose, features were extracted from the thrust force, torque and vibration signals. The following conclusions are drawn from this work:

- Cutting force signals, i.e., thrust and torque have been studied in the time domain, and vibration signals from the workpiece are studied in the time domain as well as the time frequency domain. The values of the extracted features increase for a continuing drilled hole and become the maximum before breakage.
- Prediction of drilled hole quality is better by considering all the appropriate features collectively rather than when they are considered individually. The results establish that the fusion of sensor signal features can produce higher accuracy of tool-condition prediction.
- Hole-quality prediction by ANFIS model reveals the best performance among the different monitoring strategies it is fusion of all the time signal feature. The wavelet packet-based approach is more accurate in prediction of hole quality as compared to time based approaches of vibration signals. Moreover, the different monitoring strategies are found to perform better in fusion of all sensor signal.

## Figures and Tables

**Table 1:** Experimental cutting conditions.

Cutting Condition (CC)	Drill Diameter, *D* (mm)	Spindle Speed, *n* (rpm)	Feed Rate, *f_r_* (mm/rev)	No. of Hole
CC1	0.4	10,186	0.003	44
CC2	0.4	14,186	0.002	79
CC3	0.4	19,099	0.001	41
CC4	0.4	19,099	0.003	11
CC5	0.4	14,186	0.003	24

**Table 2:** Adaptive neuro fuzzy inference system (ANFIS) performance for different combinations of time domain features.

Input Signal	Mean Training Error	Mean Testing Error
Thrust (*F_z_*)	0.10418	0.30836
Torque (*M_z_*)	0.11599	0.28671
Vibration (*A_z_*)	0.083327	0.10583
*F_z_* and *M_z_*	0.083997	0.5762
*F_z_* and *A_z_*	0.071963	0.062799
*M_z_* and *A_z_*	0.07369	0.18667
*F_z_*, *M_z_* and *A_z_*	0.063938	0.07037

**Table 3:** Performance comparison of wavelet features and time feature by ANFIS model.

Input Parameters (Z Direction Vibration Signal)	ANFIS Model	
	Mean Training Error	Mean Testing Error
Wavelet features of vibration (C34, C35, C37)	0.054371	0.080652
Time domain feature, rms vibration (A_z_)	0.083327	0.10583
